# Risk of cardiovascular events in men treated for prostate cancer compared with prostate cancer-free men

**DOI:** 10.1038/s41416-019-0468-8

**Published:** 2019-05-08

**Authors:** Ida Rask Moustsen, Signe Benzon Larsen, Anne Katrine Duun-Henriksen, Anne Tjønneland, Susanne K. Kjær, Klaus Brasso, Christoffer Johansen, Susanne Oksbjerg Dalton

**Affiliations:** 1 Survivorship, Danish Cancer Society Research Center, Copenhagen, Denmark; 2 Copenhagen Prostate Cancer Center, Department of Urology, Rigshospitalet, Copenhagen University Hospital, Copenhagen, Denmark; 3 Statistics and Pharmaco-epidemiology, Danish Cancer Society Research Center, Copenhagen, Denmark; 4 Diet, Genes and Environment, Danish Cancer Society Research Center, Copenhagen, Denmark; 5 Department of Public Health, University of Copenhagen, Copenhagen, Denmark; 6 Virus, Lifestyle and Genes, Danish Cancer Society Research Center, Copenhagen, Denmark; 7 The Oncology Clinic, Finsen Center, Rigshospitalet 5073, 2100 Copenhagen, University of Copenhagen, Copenhagen, Denmark; 8 Department of Oncology, Zealand University Hospital Naestved, Naestved, Denmark

**Keywords:** Cancer epidemiology, Heart failure

## Abstract

**Background:**

The effect of lifestyle, anthropometry and cardiovascular risk factors on cardiovascular disease in men with prostate cancer (PCa) remains unclear.

**Methods:**

Using a population-based cohort of 25,436 Danish, cancer-free men aged 50–64 years, we obtained information on self-reported pre-cancer lifestyle, objectively measured anthropometry and cardiovascular risk factors, and linked them to national health registers for information on major cardiovascular outcomes. We assessed hazard ratios (HRs) of incident acute myocardial infarction (MI), ischaemic stroke (IS) and heart failure (HF) among 1546 men diagnosed with PCa treated with first-line active surveillance, watchful waiting, intended curative or palliative treatment compared with PCa-free men during 18 years of follow-up.

**Results:**

Men who received first-line palliative treatment had higher rates of IS and HF with adjusted HRs of 2.09 (95% CI 1.49–2.93) and 2.05 (95% CI 1.43–2.94), respectively, compared with PCa-free men. The risks were increased from start of treatment. We did not find the same relation for men in any other treatment group. No differences between men treated for PCa and cancer-free controls were observed for MI after adjustment for lifestyle, anthropometry, and cardiovascular risk factors.

**Conclusion:**

Pre-diagnosis lifestyle, anthropometry or cardiovascular risk factors did not explain the risk of IS and HF in PCa patients receiving palliative treatment. The results emphasise the need for balancing disease management and monitoring of cardiovascular health in this patient group.

## Background

Since the 90s, the incidence of prostate cancer (PCa) has increased threefold in Denmark as it has elsewhere.^1–3^ This is mainly caused by better screening and the majority of men are diagnosed with localised and potentially curable disease resulting in good survival prospects for most patients.^4,5^ Currently, more than 33,000 Danish men are living with PCa comprising the largest group of male cancer survivors.^1^ Among these, a number will experience disease progression and face the consequences of cancer-targeting treatments.

A well-investigated late effect of PCa treatment is cardiovascular (CV) disease. The suggested biological mechanisms include a direct cardio-toxic effect of hormone manipulation or an indirect CV effect through metabolic mechanisms.^6–13^ Review of the literature show inconsistent results on the association between PCa and risk of CVD, which may relate to differences in age, stage, length of follow-up, type of therapy and choice of CV outcomes, which vary widely across studies. Further, few studies have compared the risk of CV outcomes for PCa patients with that of PCa-free men and with different results.^14,15^ Armenian et al.^14^ found a lower CV risk (incidence rate ratio (IRR), 0.89; 95 % confidence interval (CI) 0.84 – 0.95) compared with cancer-free men, whereas O’Farrell et al.^15^ found an increased risk, hazard ratio (HR) 1.21 (95 % CI 1.18–1.25).

When investigating the risk of CV events after diagnosis, it is paramount to address pre-diagnostic lifestyle and pre-existing CV risk to elucidate the complex relationship between existing risk factors at time of diagnosis and initiation of anti-cancer treatment and treatment-induced morbidity.^16^ Smoking, high-risk drinking and a sedentary lifestyle are factors associated with the risk of CV disease, as are a high body mass index (BMI) and waist circumference, as well as hypertension, hypercholesterolaemia and diabetes.^17–20^

We aimed to investigate risk differences of major ischaemic disease outcomes—acute myocardial infarction (MI), ischaemic stroke (IS) and heart failure (HF)—for men with PCa in either active surveillance, watchful waiting, intended curative or palliative first-line treatment compared with men without a PCa diagnosis. Analyses were adjusted for pre-diagnosis lifestyle, anthropometry, blood pressure, serum cholesterol and diabetes, as well as CV precursor disease.

## Methods

### Study population and data collection

Our study was based on data from the Danish prospective Diet, Cancer and Health cohort. Briefly, 80,996 men aged 50 – 64 years without previous cancer diagnoses were invited and 27,179 (34%) participated. Baseline information was collected between December 1993 and May 1997, where all participants completed questionnaires concerning diet, lifestyle- and health-related issues, and trained staff obtained blood samples and anthropometric measurements. A detailed description of the cohort is provided elsewhere.^21^ The Diet, Cancer and Health study was approved by the regional ethical committees in Copenhagen and Aarhus ((KF)11–037/01), and the present study by the Danish Data Protection Agency (2013–41–4232).

### Identification of PCa patients

We used the unique personal identification number provided to all Danish residents to link the cohort to the Danish Cancer Registry with records of all cancers diagnosed in Denmark.^22,23^ We identified 1838 men from the cohort diagnosed with incident PCa (ICD-10: C619) between study enrolment and 31 December 2013. By thoroughly reviewing hospital records, we obtained clinical information on stage, Prostate Specific Antigen (PSA) level, Gleason score and first-line treatment for all men diagnosed with PCa from 1993 to 2013. Stage was reported by the TNM classification system in the hospital records and was supplemented with information from the Danish Cancer Registry when missing in the record. To include stage of disease recorded before the introduction of TNM in 2003, we merged TNM with the previously used classification system, resulting in ‘localised disease’ and ‘non-localised disease’ (algorithm in Appendix). Gleason score and PSA level at baseline were categorised and included solely for descriptive purposes. First-line treatment was coded as follows: ‘active surveillance’ (surveillance with a curative intent in case of progression), ‘watchful waiting’ (surveillance with palliative intent in case of progression), ‘intended curative’ (radical prostatectomy, radiotherapy or brachytherapy) or ‘palliative treatment’ (hormone therapy (received by 94 % in this group), chemotherapy, surgery or radiotherapy) for all PCa diagnosed from 1993 to 2013. Detailed description of the clinical information was previously reported.^24^

### Outcomes

The outcomes were incident acute MI, IS or HF. Date of each incident CV event was obtained from the Danish National Patient Registry^25^ (NPR) including all in-patient hospital admissions since 1977 and outpatient contacts since 1995. We supplemented information on CV events with information from the Danish Cause of Death Registry dating back to 1970, to include fatal first-time CV events.^26^

### Covariates obtained at baseline

From self-reported questionnaires, we categorised education as ‘short’ (basic school only; < 7 years), ‘medium’ (high school or vocational; 7 – 12 years) or ‘long’ (higher education; > 12 years). Smoking was included as ‘never’, ‘former’ or ‘current’. Alcohol intake (g/day) was included as a continuous covariate. To measure physical activity, we calculated metabolic equivalent of task (MET) from an average of summer and winter physical activity multiplied by number of hours per week. MET-score was grouped in quartiles. From the anthropometric measures obtained by the staff at enrolment, the BMI was grouped as normal (<25), overweight^25–30^ or obese (>30), whiereas waist circumference in centimetre was grouped in quartiles. Diastolic and systolic blood pressure (mmHg) and serum cholesterol (mMol/L) measured at enrolment were included as continuous covariates. Blood pressure was measured at the right arm using automatic devices: TAKEDA UA 751 and UA 743 in lying position after a minimum of 5 min rest. Non-fasting total cholesterol (mMol/L) was determined in whole blood using a Lipotrend® C device with Lipotrend test strips (Boehringer Mannheim).

Information on diabetes and precursor CV disease was obtained from the NPR at baseline. Diabetes was included as a yes/no covariate. The three CV precursor diseases, angina pectoris, transitional cerebral ischaemia (see definition in Supplementary Information online) and cardiomyopathy, were coded as a combined yes/no covariate, with yes indicating the presence of one or more precursor diseases.

### Exclusions and censoring

**Fig. 1:**
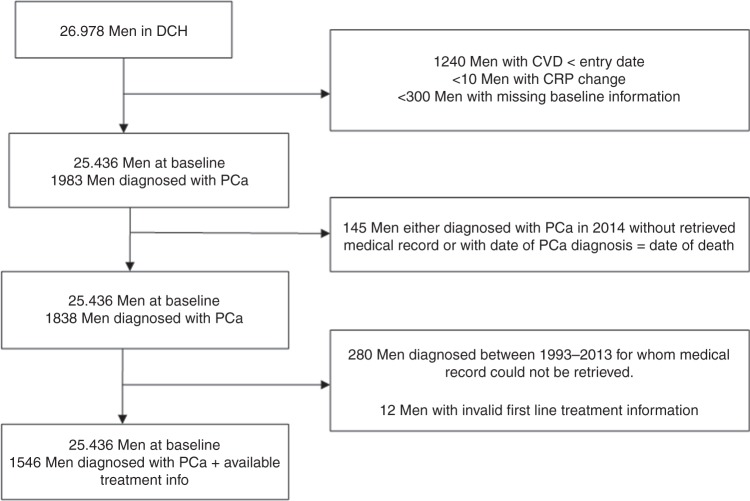
Flow diagram showing the number of study participants in the Diet, Cancer and Health cohort, and the final study population in the study, 1993–2013

Men registered with MI, IS or HF before the date of study entry (*n* = 1240, 5%) and men who changed personal identification number during follow-up (*n* < 5, <1%) or had missing values on any of the covariates of interest (*N* < 300, 1%) were excluded, leaving 25,436 men available for analysis (Fig. 1). We obtained vital status and emigration information from the Central Population Register. We were unable to retrieve information from hospital records for 280 men diagnosed with PCa and these men were not included in the study (15%).

### Statistical methods

**Fig. 2:**
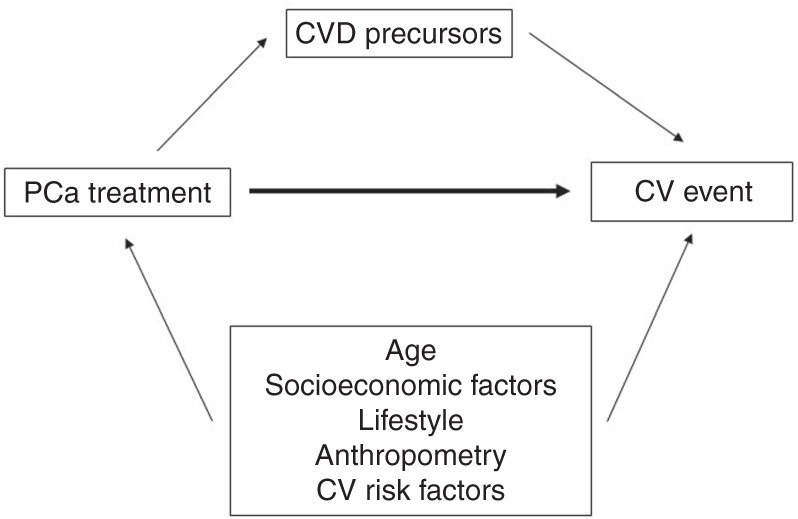
Hypothesised causal relations between lifestyle, anthropometry, sociodemographics, cardiovascular risk factors and precursors, first-line PCa treatment and CV events

First, hypothesised causal relations between confounders, mediators and CV event were identified in a diagram (Fig. 2). We then evaluated associations between these a priori determined confounders, mediators and the risk of first MI, IS or HF in the entire cohort (independent of PCa) using univariate Cox proportional hazard models. We used age as underlying timescale and included death as competing risk along with sub-distribution hazards. The age-adjusted HRs for each potential confounder was reported along with the number of men, events, deaths and person-years for the 25,436 men in the cohort.

We followed all men from date of enrolment into the Diet, Cancer and Health study to the CV outcome of interest (MI, IS or HF) or a censoring event (date of diagnosis of a competing CV event, emigration, non-CV death or end of study (31 December 2014)), whichever occurred first. First-line treatment was included as a time-varying exposure and was analysed using Cox proportional hazards models with stepwise adjustment for confounding variables, i.e., lifestyle, anthropometry and CV risk factors. Model 1 was adjusted for year of study entry. In Model 2, we added education, smoking, alcohol intake and physical activity. Model 3 further included anthropometry (BMI and waist circumference) and Model 4 included systolic and diastolic blood pressure, serum cholesterol and diabetes. In each analysis, the two other CV events were included as competing events together with death from other causes.

We estimated the cause-specific HR of each CV event stratified by first-line treatment among men with PCa compared with men without PCa adjusted for lifestyle, anthropometry and CV risk factors. We analysed temporal variation in HRs by splitting the time scale into three intervals according to time since diagnosis. As a sensitivity analysis, we adjusted for CV precursor diseases in Model 4 to see whetherpre-existing CV comorbidity confounded any association between PCa and CV events. We analysed the sub-distribution hazards for each CV event without the two other events as competing events to see whether the direction of the association remained the same.

We tested the proportional hazards assumption in all analyses by inspecting *p*-values and plots of each covariate in all models based on scaled Schoenfeld residuals. We stratified all analyses by smoking status, due to continuous violation of the proportional hazards assumption. For the covariates BMI and MET, the proportionality assumption was violated in some of the models. These models were extended by including separate baseline hazards for each level of these confounders. However, the estimates for the exposures did not change after doing this and to be consistent throughout the analyses we used the original specification of the models with one baseline hazard. Cumulative incidences were calculated for each CV outcome and death from other causes (competing event) for each category of first-line treatment. The analyses were done in R version 3.3.3 with packages ‘survival’, ‘ggplot2 and ‘etm’.^27–29^

## Results

Of the 25,436 men participating in the Diet, Cancer and Health study, 1838 (7%) were diagnosed with PCa during the median 18.5 years of follow-up accruing 387,032 person-years at risk. Only 342 (1%) men were lost to follow-up due to migration. We observed 3% of the incident 1948 MI, 4% of the 2144 IS and 4% of the 1547 HF among men diagnosed with PCa (Table 1). In the overall cohort, age-adjusted HRs for an incident CV event were increased for adverse exposure to lifestyle, anthropometry and CV risk factors (Table 1). The majority (>85%) of men treated with curative intent were diagnosed with localised disease, which was only the case for about 20% of men receiving palliative care (Table 2). Further, more than half the men in palliative care had Gleason score >8 and 75% had a PSA level above 20 at time of diagnosis (Table 2). Among the 1546 PCa patients with available treatment information, 9% were in active surveillance and 15% in watchful waiting, whereas 45% had received treatment with curative intention and 31% received palliative care as first-line treatment. We observed increased HRs for IS and HF among men in palliative treatment (adjusted HRs of 2.09, 95% CI 1.49–2.93 and HR 2.05, 95% CI 1.43–2.94, respectively) (Table 3). The adjusted cause-specific HRs did not differ by time since diagnosis among men in first-line palliative treatment nor in any other treatment group. Including the CV precursors resulted in similar estimates (data not shown). In the sensitivity analysis, we analysed CV event rates without the other two outcomes as competing risks. Results showed the same direction of associations as the main analysis (data not shown). In the time split analysis, we observed that HRs for both IS and HF were increased already within 2 years of diagnosis and were so throughout follow-up. Estimates for IS increased throughout follow-up (HR 1.37, 95% CI 0.71–2.63 within 2 years post diagnosis and HR 3.03, 95% CI 1.71–5.36 beyond 5 years of diagnosis) (Table 3).

**Table 1:** Number of men in the study population, person-years at risk, number of events and age-adjusted HRs for first CV event

Baseline characteristics	No. of men	PYRS^a^	No. of first major CV events				No. of competing events	Age-adjusted HR, HF, MI or stroke
			All CV^b^	MI	Stroke	HF	Deaths	HR (95% CI)
Total	25,436	397,085	5769	2005	2240	1632	3626	–
Educational attainment								
Long (>12 years)	6116	98,280	1136	386	463	310	722	1
Medium >7–12 years)	10,627	167,328	2320	784	932	647	1431	1.21 (1.12–1.29)
Short (≤7 years)	8693	131,476	2313	835	846	675	1471	1.43 (1.33–1.54)
Lifestyle								
Units of alcohol per week								
1–21 Drinks	13,646	214,495	3115	1158	1174	844	1843	1
22+ Drinks	8753	133,985	2067	633	842	630	1435	1.10 (1.04–1.16)
Abstainers	3037	48,678	587	214	225	158	346	1.16 (1.06–1.27)
Smoking								
Never	6699	110,614	1217	400	506	331	594	1
Former	8695	139,721	1803	623	681	533	990	1.11 (1.04–1.20)
Current	10042	146750	2749	982	1054	768	2040	1.74 (1.63–1.86)
Physical activity								
MET-score in quartiles^c^								
>43.5	6273	99,786	1424	488	562	398	928	1
>25.5–43.5	6332	102,348	1355	468	535	376	888	0.94 (0.87–1.01)
13.5–25.5	6473	99,439	1397	522	508	406	864	0.99 (0.92–1.06)
≤13.5	6358	95,511	1593	527	636	452	944	1.19 (1.10–1.27)
Anthropometry								
BMI								
Normal weight (<25)	9017	143,399	1663	555	733	402	1371	1
Overweight (25–30)	12,685	199,072	2964	1079	1134	806	1661	1.27 (1.20–1.35)
Obese (>30)	3734	54,613	1142	371	374	424	592	1.85 (1.72–2.00)
Waist circumference in quartiles								
<96	7038	110,232	1478	489	634	378	1141	1
96–100	6637	105,003	1433	527	550	383	880	1.02 (0.95–1.09)
>100–104<	5799	91,115	1296	478	518	322	765	1.07 (0.99–1.15)
>105	5962	90,734	1562	511	539	549	838	1.32 (1.23–1.41)
Cardiovascular risk factors at baseline								
Systolic blood pressure (mmHg)								
<120	2684	44,540	387	143	157	94	336	1
120–139	9581	155,179	1763	661	648	488	1246	1.28 (1.15–1.43)
140–160	9041	139,665	2189	764	840	625	1325	1.69 (1.51–1.88)
>160	4130	57,700	1430	437	596	425	717	2.59 (2.32–2.90)
Diastolic blood pressure (mmHg)								
<80	7665	124,473	1278	469	481	344	1059	1
80–89	9824	155,129	2169	794	799	620	1336	1.35 (1.26–1.45)
90–100	5682	85,663	1556	510	637	438	844	1.76 (1.63–1.89)
>100	2265	31,819	766	232	324	230	385	2.37 (2.16–2.59)
Serum cholesterol (mMol/L)								
<5	4315	67,868	863	227	359	295	688	1
5–6.5	14,053	221,436	3061	1072	1190	850	1935	1.07 (0.99–1.15)
> 6.5 < 7.5	4965	76,332	1250	468	477	331	690	1.27 (1.16–1.38)
>7.5	2103	31,448	595	238	215	156	311	1.46 (1.31–1.62)
Diabetes								
No	25,087	392,868	5599	1959	2178	1568	3558	1
Yes	349	4216	170	46	63	64	66	2.89 (2.47–3.38)
Cardiovascular precursors at baseline^d^								
0	24,846	389,299	5488	1901	2163	1530	3538	1
1+	590	7785	281	104	78	102	86	2.44 (2.16–2.75)

Number of men in the study population, person-years at risk, number of events and age-adjusted HRs for first CV event (MI, stroke, HF) with prostate cancer, education, lifestyle factors, anthropometric measures and cardiovascular risk factors and precursors in the 25,436 men in the Diet, Cancer and Health study. *CV events* cardiovascular events, *HF* heart failure, *HR* hazard ratio, *MI* acute myocardial infarction

^a^PYRS only calculated for men with available treatment information (*n* = 1546) and all controls (*n* = 25,436)

^b^Total no of CV events does not equal the sum of MI, strokes and HF reported in the table, but is based on an algorithm where myocardial infarction overrules the other outcomes and stroke overrules heart failure

^c^The metabolic equivalent of task, i.e., a measure of energy cost of physical activities

^d^Angina pectoris, transitional cerebral ischaemia or cardiomyopathy

**Table 2:** Number of men in the study population, number of person-years and hazard rates

	No. of men/ PYRS	No. of CVD events				Age-adjusted HR				Multivariable^a^ adjusted HR			
						HR (95% CI)				HR (95% CI)			
		MI	IS	HF	Death	MI	IS	HF	Death	MI	IS	HF	Death
First-line treatment^b^ (*n* = 1546)													
Men without prostate cancer	25,436/387,032	1948	2144	1537	3223	1	1	1	1	1	1	1	1
Active surveillance	133/610	6	<5	<5	<5	1.60	0.72	0.84	0.56	1.83	0.25	1.04	0.67
						(0.72–3.58)	(0.57–0.91)	(0.27–2.59)	(0.21–1.48)	(0.82–4.09)	(0.03–1.76)	(0.33–3.22)	(0.25–1.80)
Watchful waiting	230/1154	13	<10	<15	18	1.62	0.62	1.00	0.91	1.72	0.63	1.10	0.94
						(0.94–2.81)	(0.29–1.30)	(0.55–1.82)	(0.58–1.44	(0.99–2.99)	(0.30–1.33)	(0.61–2.01)	(0.60–1.49)
Curative intended treatment	695/4771	18	25	26	57	0.61	0.67	0.87	1.00	0.69	0.75	1.04	1.11
						(0.38–0.97)	(0.45–1.00)	(0.59–1.28)	(0.78–1.30)	(0.44–1.11)	(0.51–1.12)	(0.70–1.53)	(0.85–1.43)
<2 Years		4	2	6	–	0.51 (0.19–1.37)	0.22 (0.06–2.05)	0.92 (0.41–2.04)	–	0.58 (0.22–1.54)	0.25 (0.06–0.98)	1.07 (0.48–2.38)	–
2–5 Years		7	12	8	–	0.68 (0.33–1.44)	0.94 (0.53–1.66)	0.81 (0.41–1.63)	–	0.77 (0.37–1.62)	1.04 (0.59–1.84)	0.95 (0.47–1.91)	–
>5 Years		7	11	12	–	0.61 (0.29–1.29)	0.71 (0.39–1.29)	0.90 (0.50–1.57)	–	0.71 (0.33–1.50)	0.82 (0.46–1.50)	1.08 (0.61–1.92)	–
Palliative treatment^c^	488/1983	8	35	31	226	0.63	2.06	2.09	7.79	0.63	2.09	2.05	7.78
						(0.32–1.27)	(1.47–2.88)	(1.46–2.98)	(6.78–8.94)	(0.31–1.26)	(1.49–2.93)	(1.43–2.94)	(6.78–8.94)
<2 Years		2	9	11	–	0.38 (0.10–1.54)	1.36 (0.71–2.62)	1.97 (1.09–3.56)	–	0.38 (0.09–1.51)	1.37 (0.71–2.63)	1.93 (1.06–3.49)	–
2–5 Years		3	14	12	–	0.66 (0.21–2.05)	2.26 (1.33–3.82)	2.21 (1.25–3.90)	–	0.66 (0.21–2.05)	2.31 (1.36–3.91)	2.12 (1.25–3.91)	–
>5 Years		3	12	8	–	1.04 (0.34–3.25)	2.86 (1.62–5.07)	2.09 (1.04–1.19)	–	1.08 (0.34–1.50)	3.03 (1.71–5.36)	2.10 (1.04–4.22)	–

Number of men in the study population, number of person-years and hazard rates (with accompanying 95 % confidence intervals) for acute myocardial infarction, ischaemic stroke and heart failure for men with PCa in active surveillance, watchful waiting, curative intended treatment and palliative care compared with men without prostate cancer. HRs for CV events within the first 2 years, 2–5 years and more than 5 years post treatment with either curative intent or palliative care are shown. *HF* heart failure, *IS* ischaemic stroke, *MI* acute myocardial infarction

^a^Adjusted for year of entry, age, education, smoking, alcohol and physical activity, anthropometry and CVD risk factors

^b^Available for 85% of the men diagnosed with prostate cancer from 1993 to 2013 with available first-line treatment information

^c^>90% of these men received hormone therapy

All estimated HRs are cause specific

**Table 3:** Clinical characteristics for the 1546 men who were diagnosed with PCa from 1993 to 2013, who participated in the Diet, Cancer and Health study

	Watchful waiting *n* = 230		Active surveillance *n* = 133		Curative intended treatment *n* = 695		Palliative care *n* = 488	
Stage at diagnosis	*N*	%	*N*	%	*N*	%	*N*	%
Localised	213	94	127	>95	603	>85	113	>20
Non-localised	13	6	<5	<5	68	10	366	75
Missing	<5	<1	<5	<1	24	<5	9	<5
Gleason score								
6 or lower	117	51	104	78	245	35	28	6
7	77	33	20	15	264	38	127	26
8 or higher	12	5	<5	<1	133	19	264	54
Missing	24	10	<10	<10	53	8	69	14
PSA level at diagnosis								
10 or lower	131	57	106	80	357	51	34	<10
Between 10–20	64	28	25	19	204	29	70	14
20 or higher	27	<15	<5	<1	116	17	365	75
Missing	8	<5	<5	<1	18	3	19	<5

**Fig. 3:**
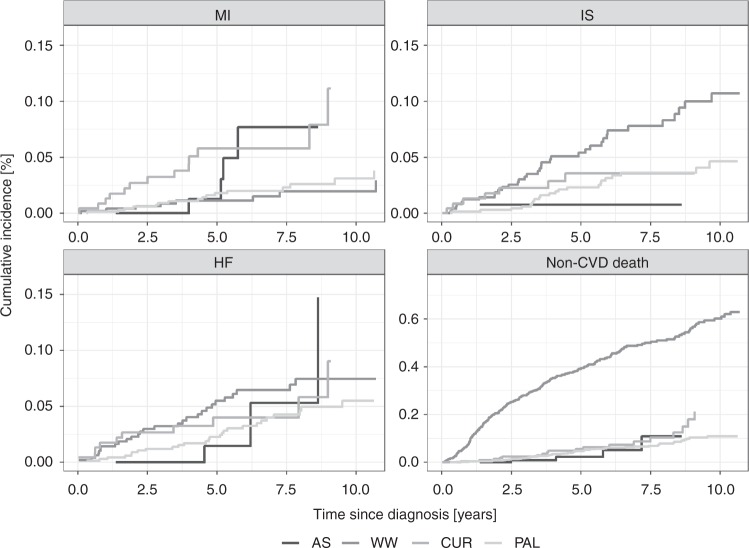
Cumulative incidence plots for acute myocardial infarction, ischaemic stroke and heart failure, and death from other causes as competing event over time since diagnoses among 1546 men diagnosed with prostate cancer from 1993 through 2013

Cumulative incidence curves of CV events and non-CV death showed that the specific distribution by CV event varied (Fig. 3). The most pronounced difference was found for IS beyond 2.5 years post diagnosis in men receiving first-line palliative care.

## Discussion

We observed a twofold higher rate of both IS and HF among men treated with first-line palliative treatment for PCa compared with PCa-free men. The risk was increased since initiation of first-line treatment and for stroke we observed an increase in the HR rate over time. Adjusting for pre-cancer lifestyle factors, anthropometry and CV risk factors did not affect the increased risk. Among men treated curatively or conservatively for their PCa, we found no statistically significant difference in risk for either MI, IS or HF compared with men without PCa.

The increased rate for HF in men receiving first-line palliative treatment for PCa compared with cancer-free men has also been observed in a population-based Swedish study. In this study, men with incident PCa undergoing endocrine treatment (i.e., antiandrogens (AAs), orchiectomy, gonadotropin-releasing hormone (GnRH) agonists or combined AA/GnRH agonist treatment) had an increased standardised incidence rate of 1.66 (95% CI 1.57–1.76) for HF compared with that expected based on the Swedish male background population without pre-existing circulatory disease.^30^ Unlike our study, they also found an increased risk of HF in the men who received curative treatment. Another Swedish matched cohort study compared the risk of HF between 41,362 ADT-treated PCa patients and age-matched controls. The HR was 1.22 (95% CI 1.14–1.31) among men who received GnRH agonists and 1.12 (95% CI 1.04–1.21) among men who underwent surgical orchiectomy at time of diagnosis, but 0.83 (95% CI 0.71–0.97) among those receiving AA, when adjusting for history of CV disease, education, stage and statin use. This study used PCa-free men as reference group; however, this did not exclude men with pre-existing CV disease and they did not include lifestyle factors in the analyses.^15^ Regarding IS, we found a twofold increased rate among men managed on palliative care compared with PCa-free men, and to our knowledge the risk of IS in PCa patients has never been compared with the risk in men without PCa. One Danish cohort study including >30,000 men diagnosed with PCa observed an increased, although much lower adjusted HR for stroke of 1.19 (95% CI 1.06–1.35) among men managed on endocrine therapy (GnRH agonists, AA) but not in those undergoing orchiectomy compared with non-users of Androgen Deprivation Therapy (ADT).^16^ A US study of 22,310 men with non-metastatic PCa reported an increased risk of stroke or transient ischaemic attack (TIA) in men undergoing bilateral orchiectomy (Relative risk (RR) 1.77, 95% CI 1.25–2.39) compared with non-ADT users, with patients age >65 years at diagnoses having an increased risk for stroke/TIA compared with the younger patients.^31^ We did not find an increased risk of MI in any treatment group when comparing with PCa-free men, which contrasts to large PCa cohort studies from United States and the United Kingdom including a minimum of 30,000 PCa patients and reporting risk estimates in the range of 1.09 to 1.40 (95% CI 1.01–1.49).^30,32,33^ To our knowledge, only one study has compared the risk of ischaemic heart disease, stroke and cardiomyopathy/HF between PCa patients and cancer-free men.^14^ The study found that among 10,932 PCa patients who survived diagnosis by 2 years, those with American Joint Committee on Cancer (AJCC) stage I/II and III had a lower CV risk (IRR 0.89, 95% CI 0.84–0.95 and IRR 0.79, 95% CI 0.66–0.95, respectively) compared with cancer-free men. The risk did not differ between PCa patients with stage IV and cancer-free men (IRR 1.03, 95% CI 0.78–1.35).^14^ However, the study lacked information on pre-diagnosis lifestyle, comorbidity and PCa treatment, and the study population was selected based on participants in an intensive screening programme through insurance coverage, which might have introduced selection bias as well as a high percentage of advanced tumours.

The strengths of our study include the use of high-quality registers. The Danish Cancer Registry holds accurate and almost complete recording of incident cancers^23^ and The NPR include information on both in- and outpatient contacts for CV events, which has been validated for research use.^34,35^ Further information on first-line treatment of PCa cases was obtained independently of the outcome of interest. We included all cancer stages in our analyses, thus providing a comprehensive perspective on how PCa treatment is associated with the risk of CV outcomes. Furthermore, our study had several methodological strengths. We made the comparison of men with PCa with a cohort of cancer-free men to disentangle the role of shared risk factors for PCa and CV disease. To the best of our knowledge, we are the first to include prospectively collected high-quality information on pre-cancer lifestyle, objectively measured body composition, CV risk factors and precursor heart disease in the same study. We include both competing risk analysis and sub-distribution hazards to account for the significant competing events present in this study population. In our study, the results from sub-distribution hazard analyses aligned with the cause-specific hazards, which strengthens our interpretation that the estimated rates are likely to reflect a direct effect of PCa and treatment on the studied CV events.^36^ Our study has some limitations regarding information on outcomes of interest, treatment and selection into the study. Most cases of MI and IS will cause admission or other hospital contact. However, we acknowledge that our study results may still be prone to surveillance bias, i.e., that men diagnosed with PCa may have a higher probability of being diagnosed with particularly HF. When diagnosed with PCa, assessment of their stage of disease, symptom burden and severe comorbidity may result in confounding by indication. We excluded all men with pre-existing events, but still saw indications, albeit statistically nonsignificant of higher risk of MI among conservatively treated men and lower risk of MI among curatively treated men. No first-line treatments were associated with changes in risk over time after diagnosis. Among palliative treated men, the risk for both IS and HF was increased throughout the full follow-up period, despite a high non-CVD mortality, indicating that an effect of palliative first-line treatment on these CV outcomes may present from beginning of treatment and pointing to a need for early and targeted surveillance among these men. PCa patients initially managed on either active surveillance or watchful waiting may later have changed treatment strategy, and likewise men undergoing curatively intended therapy may subsequently have progressed during follow-up, potentially underestimating the difference between men who receive first-line palliative treatment and those who receive other first-line treatments. Watchful waiting is in general associated with older age, more comorbidity and shorter life expectancy than other treatments. This may explain the persistent findings among the conservatively treated men in watchful waiting of an increased, albeit statistically nonsignificant, risk of MI, whereas the risk for IS and HF tended to be very close to null or even reduced. As we excluded men with pre-existing CV events, this may have introduced more selection in terms of health in the group of patients allocated to watchful waiting than in the other treatment groups.

Some studies suggest that the risk of subsequent CV events correlates to the type of hormone therapy;^16,37^ however, we were not able to discriminate between subtypes of hormone therapy in the available data. This could efface within-group differences in the palliative group due to an underestimation for those receiving GnRH agonists and an overestimation among those who underwent bilateral orchiectomy. Contrary to expectations, the stepwise adjustment for lifestyle, anthropometry and CV risk factors did not affect the effect estimates markedly indicating that men with PCa in this cohort do not differ from the comparable men in terms of these CV risk factors. This may be because the men in the Diet, Cancer and Health cohort are somewhat healthier than the general population, thus resulting in less variation in risk factors, which is a well-known phenomenon in large-scale population-based cohorts.^38–40^

We are aware that the results from this study population may not be generalisable to populations that have different baseline CV risks than Danish men and in settings with new chemo- and endocrine regiments available. Recent studies have demonstrated that combined chemo- and endocrine therapy,^41^ and addition of abiraterone to standard hormone therapy^42^ can improve survival in men with newly diagnosed metastatic PCa. The patients included in the present study were diagnosed through December 2013 and in the present period only few patients had chemotherapy in combination with first-line endocrine treatment. Abiraterone and second-generation AAs, i.e., enzalutamide and apalutamide, have not yet been approved in Denmark in combination with standard endocrine therapy in men with newly diagnosed metastatic disease. Thus, our data cannot provide information on the role of these therapies that would be both relevant and interesting to investigate in future studies, in PCa populations in relation to CV events. We encourage interpretation of the results to other settings with this in mind.

In summary, we found an increased risk of IS and HF among men who received first-line palliative treatment compared with men without cancer after adjusting for pre-cancer lifestyle, anthropometry and CV risk factors. As many of these men live with their PCa for a long time, it remains necessary to balance symptom relief and disease control with monitoring CV health in this patient group from the upstart treatment, in order to prevent or secure early management of CV events potentially affecting overall morbidity, health-related quality of life and ultimately mortality.

## Supplementary information

Supplementary information

## Data Availability

The data used in the analyses for the manuscript is stored securely at the Danish Cancer Society Research Center and are available on request.
