# Supplementary material for: Risk of cardiovascular events in men treated for prostate cancer compared with prostate cancer-free men
Source: Br J Cancer. 2019 May 8;120(11):1067–74. doi: 10.1038/s41416-019-0468-8 (PMC6738102; doi:10.1038/s41416-019-0468-8)
Supplement: Supplementary file 1 — Supplementary information [file 41416_2019_468_MOESM1_ESM.docx]

**Staging of PCa**

If information on stage was missing in medical records, we used the Danish Cancer Registry to identify stage. Stage was classified by the TNM system and by the system prior to TNM (c_udbred) were merged into the categorical variable ‘stage’ with two levels (‘localized’ and ‘non-localized’) by the following algorithm:

If c_udbred = 5 (localized disease)

or T1-2, x and N0,x and M0

then stage = ‘localized’

If c_udbred = 6 (locally advanced disease) or 7(metastatic disease)

or T3-4, x or N1-3 or M0,x

then stage = ‘non-localized’

**Definition of cardiovascular precursors and outcomes**

ICD-8 and ICD-10 codes for the outcomes; myocardial infarction, stroke and heart failure and for the cardiovascular precursors; angina pectoris, transitional cerebral ischemia and cardiomyopathy

|  | **DIAGNOSIS** | **ICD-8** | **ICD-10** |
| --- | --- | --- | --- |
| Cardiovascular outcomes | Myocardial infarction | 41009, 41099 | I21-I22 |
|  | Stroke | 43309, 43399, 43409, 43499, 43601, 43690 | I63, I64 |
|  | Heart failure | 42709, 42710, 42711, 42719 (110,157) | I110, I130, I132, I420, I421, I50, J819 |
| Cardiovascular pre-cursors | Angina Pectoris | 41309, 41399 | I20 |
|  | Transitional cerebral ischemia | 43599 | DG45.0-4, DG45.8, DI660, DI66.2-4, DI668, DI669 |
|  | Cardiomyopathy | 74640, 74641, 74649 | DI42.X, DI43.X |
